# Scraps

**Published:** 1900-12

**Authors:** 


### SCRAPS

PICKED UP BY L. M. G.

**Clinical Records** (33).—A pale and dishevelled Frenchman was sinking into his deck-chair on a Channel steamer, when a passenger asked cheerily: "Ah, monsieur, have you breakfasted?" "No, monsieur," answered the pallid Frenchman, "I haf not breakfasted—on ze contrairy!"

**Medical Philology** (XXXVI.).—Appended to the entry in the *Promptorium* of "Dragge. *Dragetum*," Mr. Way gives the note: "The word is taken from the French *dragée*, a kind of digestive and stomachic comfits anciently much esteemed," and he quotes from Chaucer's "Doctour of Phisyk" (Prologue, 436):

> "Ful redy hadde he his apothecaries,
> To send him dragges, and his lettuaries."

But the reading "dragges" appears in only one of the old MSS. The others have "drogges" or "drugges," and the first of these is the word now usually accepted. It is most likely that Chaucer meant to refer to remedies of a more general character than would be denoted by the distinctly limited term. Cotgrave says dragée is "a kind of disgestiue powder, vsually prescribed vnto weake stomackes after meat; and hence, any ionkets, comfets, or sweet meats, serued in at the last course, (or otherwise) for stomacke-closers."

**Medical Education.**—At a recent Congress of the American Physicians and Surgeons, Dr. H. P. Bowditch, speaking of "The Medical School of the Future," said: "One of the most hopeful signs of the times in the field of medical education is the growing tendency of the better schools to ally themselves to universities and of universities to establish medical departments. . . . In fact, it is difficult to see how a private medical school of the joint stock company type can ever, in the future, rise to the first rank, for such a school is not much more likely to attract endowments than a cotton mill and without endowments the enormous expenses of a modern first-class medical school cannot possibly be met," considering that "'the immense deepening and widening of human knowledge in the nineteenth century and the increasing sense of the sanctity of the individual's gifts and will power.'" He pleads for the early specialisation of the student's work.

*The Boston Medical and Surgical Journal*, commenting on Dr. Bowditch's address, says: "We are glad to note a perfectly definite desire to do full justice to the methods of the past, while recognizing the fact that some of those methods have outgrown their usefulness. This, coupled with the conviction that medical knowledge has passed beyond the limits of a rigidly prescribed course, gives to the address a judicial quality which is at times lacking in the first enthusiasm of reform. We have of late frequently found occasion to express ourselves as advocates of change in the form of instruction. This is inevitable and wholly desirable, but this should never lead us into the error of supposing that the acquisition of knowledge is entirely dependent upon the manner of its presentation. The teacher is, after all, the essential element in the success of any system. We look, therefore, to a readjustment of the form of instruction which will lead us to the development of the teacher as well as of the student. We have no doubt that the didactic lecture will be dignified rather than degraded by the attacks made against it, and the observation and independence of the student cultivated at the same time, through individual work in a general atmosphere of greater freedom in the choice of his studies. These seem to be some of the lessons which Dr. Bowditch's address teaches."

Dr. Lewellys F. Barker, in an address "On the Present Status of Therapy and its Future," in the *Bulletin of the Johns Hopkins Hospital*, thinks that "perhaps the most significant movement at present observable in medicine is the beginning of the application of the newer ideas of physics and chemistry to the solution of biological questions." He says that the principles underlying the new methods of approaching problems are "so fundamentally important and have already worked such marvelous transformations in the mode of thought and activity of chemists that we are justified in expressing great hope for the future in their use by medical investigators," to some of the already-attained results of which he alludes. He is so confident "that the promise for the future in pathogenesis and in pharmacodynamics is much brightened by the advent of physical chemistry," that were a medical student to ask him the question, "How can I best fit myself to make real advances in knowledge in medicine and therapy during the next twenty-five years?" he would say, "In addition to a thorough medical course, arm yourself with sufficient mathematics and gain a thorough theoretical and practical training in the methods of physics and chemistry and especially in the principles and methods of what is called 'physical chemistry.' After this turn your attention to the solution of medical problems."

Dr. W. W. Keen delivered the presidential address at the annual meeting of the American Medical Association. It is printed in full in *The Medical News* of June 9th, 1900. After entering into details of the necessities of increased laboratory work in medicine, Dr. Keen said: "But, the function of the medical school should not be limited merely to the imparting of existing knowledge. No school is worthy of the name that does not provide for greater or less research work, by which some substantial additions to our knowledge may be made, and the facilities and the results of the healing art made more efficient for the welfare of mankind. . . . Our hospitals do a magnificent work in charity, helping the sick and the forlorn, the weak and the suffering, in a way which appeals to the charitable instincts of our fellow-countrymen, and to this appeal they have responded most generously. *But I venture to say that the medical school which trained a Lister, a Pasteur, a Koch, has done more for humanity than all the hospitals of this country combined.* The modest laboratory at Würzburg consisted chiefly of a Rhumkorff coil, and a Crookes' tube — and Röntgen. Other Röntgens and Listers we have among us if we but knew it. These are the men who are the world's real illustrious heroes."

The view of one who is not a medical man, but who has had large experience in teaching, deserves attention. Dr. Andrew V. V. Raymond, the President of Union College, speaking on "Education and the Profession of Medicine" before the Medical Society of the State of New York, said: "I will not say that there is no disciplinary value or no fertilizing influence in purely professional studies, but this I do say, that in these respects they do not compare with other studies which find no place in your curriculum. It will be admitted by all that what is needed for the successful prosecution of any professional course is intellectual vigor, mental grasp, ability to comprehend statements, to follow a line of reasoning, to think clearly and closely. I will venture the assertion that lack of this ability is the greatest difficulty encountered by the teachers in your medical schools, and it is not due to the natural dulness of the students, but to the absence of such preliminary training as makes a man master of his own intellectual forces, giving him the power to concentrate thought and hold his mind to a subject till it is understood."

